# The Effect of Chronic Endurance Exercise on Serum Levels of MOTS-c and Humanin in Professional Athletes

**DOI:** 10.31083/j.rcm2305181

**Published:** 2022-05-18

**Authors:** Maha Alser, Manjunath Ramanjaneya, Najeha Rizwana Anwardeen, Francesco Donati, Francesco Botrè, Jayakumar Jerobin, Ilham Bettahi, Nura Adam Mohamed, Abdul Badi Abou-Samra, Mohamed A Elrayess

**Affiliations:** ^1^Biomedical Research Center, Qatar University, 2713 Doha, Qatar; ^2^Qatar Metabolic Institute, Hamad Medical Corporation, 3050 Doha, Qatar; ^3^Translational Research Institute, Hamad Medical Corporation, 3050 Doha, Qatar; ^4^Laboratorio Antidoping, Federazione Medico Sportiva Italiana, 00197 Rome, Italy; ^5^QU Health, Qatar University, 2713 Doha, Qatar

**Keywords:** professional athletes, endurance, age, mitochondrial proteins, humanin, MOTS-c

## Abstract

**Background:**

Humanin and the mitochondrial open reading frame of the 12S rRNA-c (MOTS-c) are mitochondrial encoded peptides involved in energy metabolism, cytoprotection, longevity, insulin sensitivity and their expression decrease with age. Levels of these molecules have been shown to respond to acute exercise, however little is known about their modulation under different chronic exercise conditions. In this study, we aim to compare levels of Humanin and MOTS-c in non-athletes vs professional (moderate and high endurance) athletes.

**Methods:**

Serum samples were collected from 30 non-athlete controls and 75 professional athletes (47 low/moderate endurance and 28 high endurance athletes). Levels of Humanin and MOTS-c were measured by the enzyme linked immunosorbent aaasy (ELISA) and linear models were generated to compare the effect of different levels of endurance exercise on these factors in different age groups. Spearman correlation was used to assess the correlation between these factors in athletes and non-athletes.

**Results:**

We showed that professional athletes had lower levels of MOTS-c and higher levels of Humanin than sedentary controls. Within the athletic groups, high endurance athletes had lower levels of Humanin than low/moderate endurance athletes of the same gender/age groups, whereas MOTS-c levels did not change between the subgroups. Humanin and MOTS-c levels were highly correlated in athletes, but not in sedentary controls.

**Conclusions:**

This pilot data suggests that serum levels of the mitochondrial proteins MOTS-c and Humanin change in response to chronic exercise with implications on energy metabolism and performance.

## 1. Introduction

The mitochondria is a vital organelle involved in cellular metabolism, aging and physiological functions [1]. Humanin was the first identified mitochondrial derived 24 amino acid peptide (MDP), encoded by a short open reading frame within the mitochondrial genome. Low expression of Humanin is linked to cell senescence, inflammation, and cognitive decline [2]. As a cytoprotective factor, it improves metabolic health, and reduces inflammatory [3], and oxidative stress markers in the cells [4]. Studies have suggested its protective role in various metabolic disorders, particularly improving insulin sensitivity and preventing type 2 diabetes [4]. Studies have also suggested that Humanin plays a major role as a metabolic mediator in cardiovascular disorders as the levels of this peptide increase in the left ventricle of the cardiac muscle as a response to severe stress [4]. Other studies have shown various clinical and basic reports that suggested an anti-apoptotic effect of Humanin in the cardiac muscle and vasculature [5]. Another member of the MDPs is the mitochondrial open reading frame of the 12S rRNA-c (MOTS-c). MOTS-c is a 16 amino acid peptide, expressed mainly in the muscular tissue with low basal levels in the plasma and other tissues [6]. Like Humanin, MOTS-c plays an important cytoprotective role as its levels increase in response to stress. MOTS-c also plays a crucial role in cellular metabolism, regulating glucose homeostasis and insulin sensitivity in skeletal muscle and impeding obesity [7]. Levels of MOTS-c were also found to be associated with longevity in centenarians [8]. As MOTS-c plays a key role in regulating cardiovascular diseases (CDs) risk factors (atherosclerosis, ageing, insulin resistance, and hyperlipidemia), recent research has suggested that MOTS-c is one of the main cardioprotective mitochondrial peptides, affecting CDs development and progression [9].

Exercise improves human health at different levels. At the physiological level, exercise helps in preventing and treating obesity and other related metabolic complications [10]. Humanin levels were shown to increase after acute exercise in muscle tissue and plasma [11], however, they decline with aging [12], mainly due to the drop in total mitochondrial mass in the muscular cells [6]. MOTS-c, on the hand, was suggested to play a critical role in regulating exercise as it enhances the physical capacity of athletes [13]. MOTS-c protein expression were shown to increase post-acute exercise in human, and increase the physical capability to perform exercise in mice [1]. These findings suggest that MOTS-c levels are more likely to be affected by chronic exercise. The plasma levels of Humanin and MOTS-c have been shown to respond to acute exercise in an age-dependent manner, but little is known about effect of chronic exercise on serum levels of these proteins [11]. Here, we aim to compare the serum levels of Humanin and MOTS-c in healthy individuals with sedentary lifestyle and two groups of professional athletes (low/moderate and high endurance). We will also compare the differences in the levels of Humanin and MOTS-c expression between two age groups. Understanding the effect of different degrees of chronic exercise on the levels of these critical cytoprotective mitochondrial peptides will help us further understand the impact of chronic exercise on the overall health and performance of professional athletes.

## 2. Materials and Methods

### 2.1 Sample Collection

Blood samples of 75 professional athletes were collected by the anti-doping lab in Rome, Italy (FMSI) in the framework of doping control tests. The cohort included 63 male and 12 female professional athletes from differerent sport disciplines who participated in national and/or international sports competitions and tested negative for doping abuse by accredited antidoping labs. The cohort was classified into two different groups of sport intensity: high endurance and moderate/low endurance groups. Table 1 summarizes the sport disciplines per endurance category and the number of participants included in this study. These disciplines were categorized based on the intensity level of the dynamic component, i.e., the estimated percent of maximal oxygen uptake, and the static component, i.e., the estimated percent of maximal voluntary contraction [14, 15]. The cohort were further sub divided into two groups according to age; above 30 and below 30 years old. The cohort also included 30 sedentary controls, matched with professional athletes for age and body mass index. The non-athletes (control) group of participants did not participate in any regular exercise program. The age and gender classifications are summarized in Table 2. All procedures applied in this research were approved by the Institutional Research Boards (IRBs) of Qatar University (QU-IRB 1277-E/20) and Hamad medical corporation (MRC 16245/16), and all participants were consented prior using their samples for research purposes.

**Table 1.** **Classification of Participants from Each Sport Discipline into Endurance Groups (Low/Moderate and High Intensity)**.

Low/Moderate endurance (n = 47)	High endurance (n = 28)
1 Cricket (1 M), 1 Equestrian (1 M), 1 Golf (1 M), 1 Powerboating (1 M), 1 Sport climbing (1 M), 30 Football (30 M), 1 Athletics-throws (1 M), 2 Bobsleigh (1 M, 1 F), 2 Gymnastics (2 F), 1 Luge (1 M), 1 Volleyball (F), and 5 Wrestling (5 M)	11 athletics (1 F, 10 M), 10 Cycling (5 F, 5 M), 7 triathlon (2 F, 5 M)

M, male; F, female.

**Table 2.** **Characteristics of Study Participants. Data are Presented as n (Mean, SD) for Parametric Continuous Variables, Median (IQR) for Non-Parametric Continuous Variables. Categorical Factors are Displayed as Counts**.

		Non-athlete (n = 30)	Athlete (n = 75)	
			Low/Moderate Endurance (n = 47)	High Endurance (n = 28)
Age (years, continuous)		30 (30, 2.95)	47 (26.55, 6.97)	28 (26.54, 8.92)
Age (categorical)				
	Below 30	14 (27.43, 2.13)	30 (22.1, 2.63)	19 (21.32, 3.46)
	Above 30	16 (32.25, 1.18)	17 (34.4, 4.89)	9 (37.56, 6.34)
Gender				
	Male	18 (22%)	43 (53%)	20 (25%)
	Female	12 (50%)	4 (17%)	8 (33%)
MOTS-c (ng/mL)		3.89 (3.1, 4.88)	2.36 (1.74, 4.18)	2.22 (1.54, 3.94)
Humanin (pg/mL)		774 (676, 898)	1258 (615, 1594)	24, 1114)

MOTS-c, mitochondrial open reading frame of the 12S rRNA-c.

### 2.2 MOTS-c and Humanin Measurement

Serum MOTS-c levels were measured using a commercially available ELISA kit (BMA Biomedicals; Catalog number: S1526, Rheinstrasse, Switzerland) following manufacturer’s instructions, with a detection range between 0.10 and 100 ng/mL and intra-assay variation of less than 10% and inter-assay coefficient of variation of <12%. Serum Humanin concentrations were measured using a commercially available ELISA kit (CUSABIO, Houston, USA; Catalog number: CSB-EL015084HU) according to manufacturer’s recommended protocol, with a detection range between 28 and 1800 pg/mL and intra-assay coefficient of variation of <8% and inter-assay coefficient of variation of <10%.

### 2.3 Statistical Analysis

Measurements were normalized using cube- and log- transformations for Humanin and MOTS-c respectively. R version 4.0.3 (R Core Team, Vienna, Austria) was used to run the linear models per peptide against athlete vs controls and high-endurance athletes vs low/moderate endurance athletes, while also correcting for potential confounders including gender and age. Data are presented as median (IQR) for continuous variables whilst categorical factors are displayed as counts.

## 3. Results

### 3.1 MOTS-c and Humanin Levels in Professional Athletes Serum vs Non-athlete

**Fig. 1.**
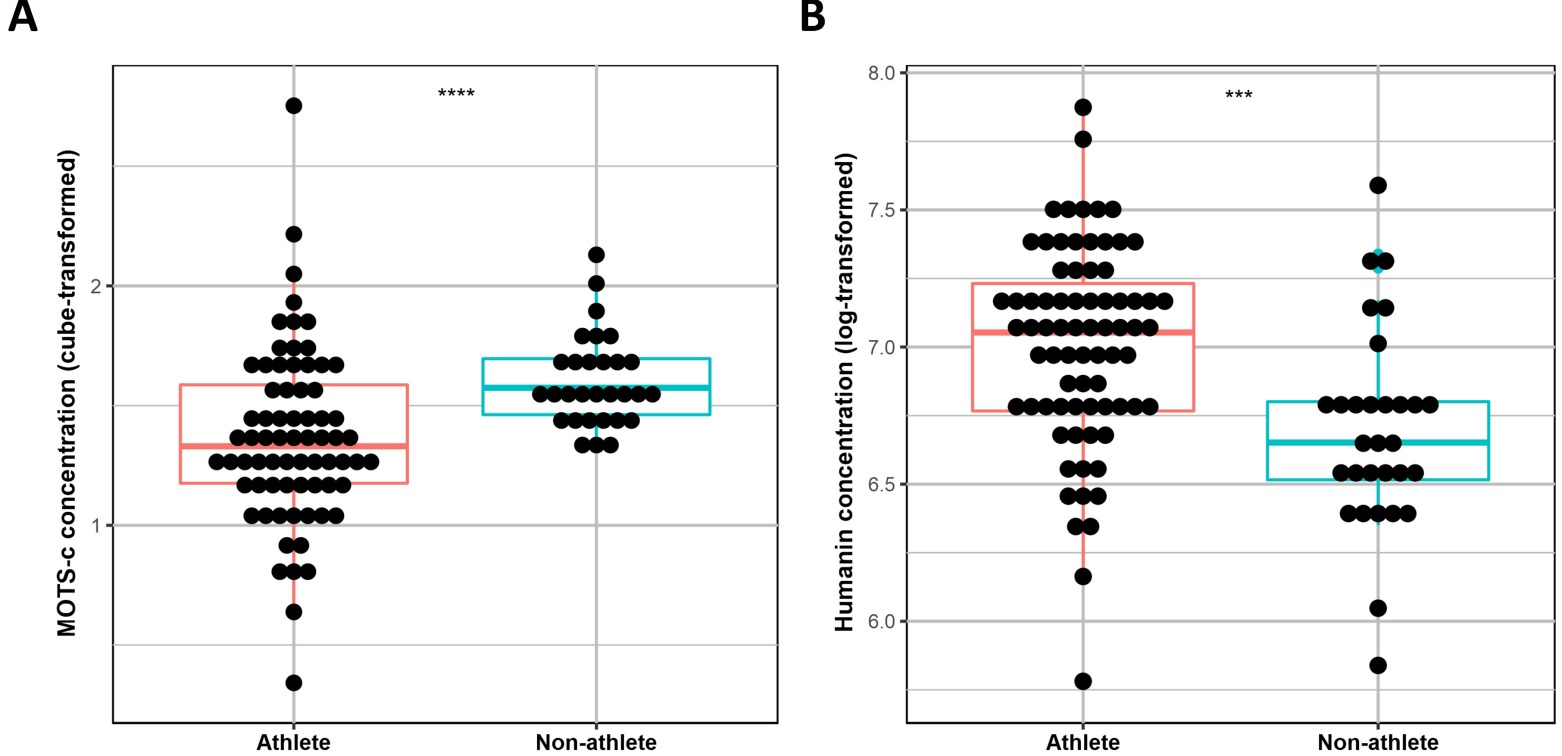
**Differences in MOTS-c (A) and Humanin (B) serum levels between Athletes and Non-Athletes**. A linear regression model was used to assess differences in these peptides between athletes and non-athletes. *** = *p *< 0.005, **** = *p <* 0.0005.

Serum levels of MOTS-c and Humanin were compared between professional athletes (n = 75) and a non-athletic group (n = 30) while correcting for age and gender. Our results showed that MOTS-c serum levels were significantly lower in the athletic group (*p* = 0.0001), while levels of Humanin were significantly increased in the athletic group (*p *= 0.005) (Fig. 1).

### 3.2. Humanin and MOTS-c Levels between Low, Moderate, and High endurance Professional Athletes

**Fig. 2.**
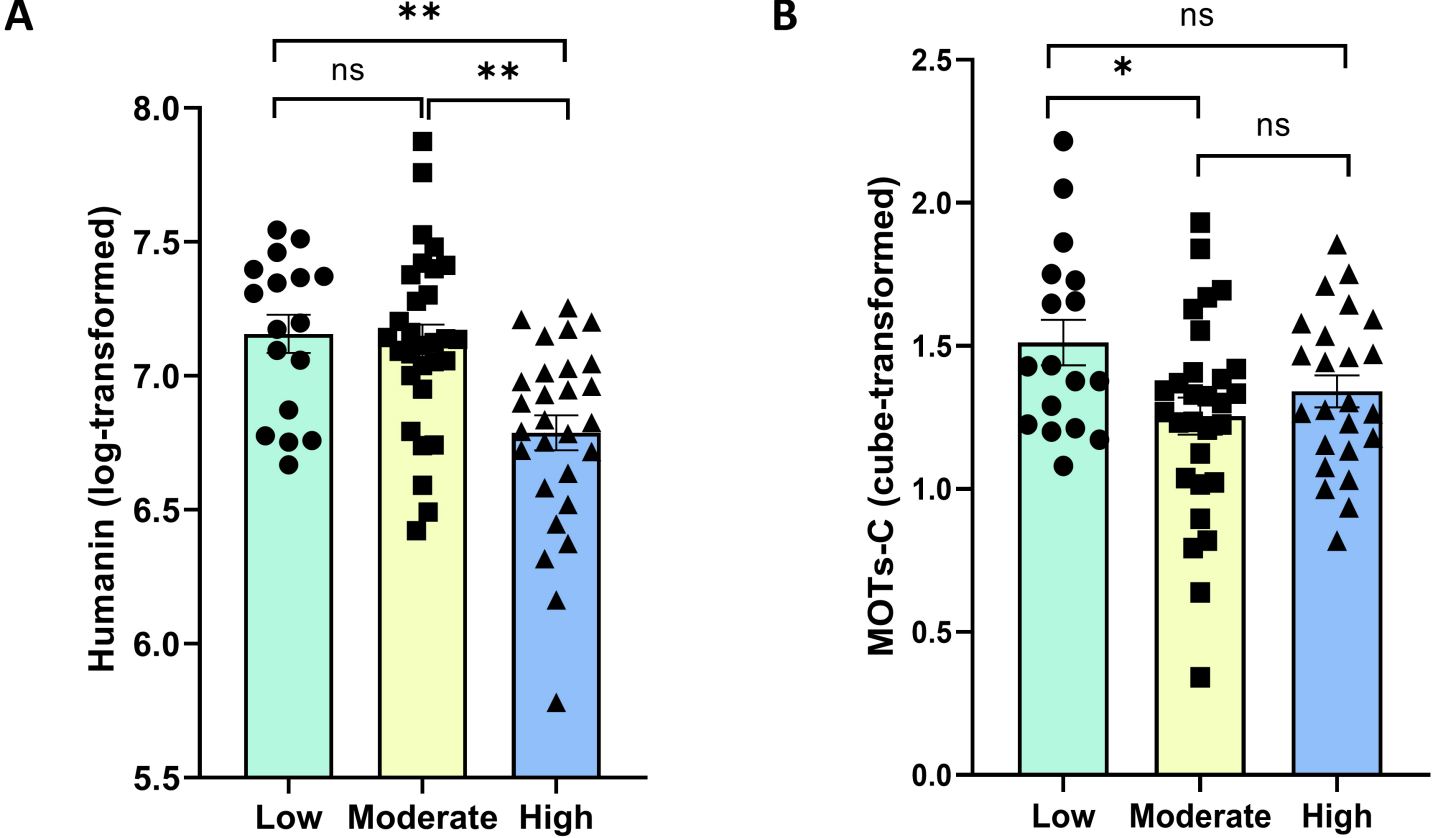
**The difference in Humanin (A) and MOTS-c (B) serum levels between low, moderate, and high endurance athletic groups**. A linear regression model was used to assess differences in these peptides between the different athletic groups. Data is presented as mean ± SEM for n = 17 low, n = 30 moderate, and n = 28 high endurance participants. Statistical analysis was determined by *t*-test. Ns = non-significant, * = *p* < 0.05, and ** = *p *< 0.01.

As differences in MOTS-c and Humanin levels between athletes and non-athletes were observed, we further investigated the difference among different athletic groups (low, moderate, and high endurance athletic groups) to understand the impact of exercise intensity on levels of MOTS-c and Humanin. Humnin was significantly lower in high endurance athletes compared to low endurance (*p *< 0.001) and moderate endurance (*p *< 0.001). While there was no observed significant difference between low and moderate groups. On the other hand, MOTS-c levels showed a dose dependent reduction as endurance level increased. The MOTS-c serum levels in low endurance group was significantly higher than the moderate endurance group (*p *< 0.05), with no significant difference between the rest of the groups as shown in Fig. 2. Because the number of participants is unequally distributed between the groups, we performed the rest of analyses combining the 2 groups (low and moderate endurance) as one group compared to high endurance group in the following sections.

### 3.3 Humanin Levels in Professional High Endurance Athletes vs Low/Moderate Athletes

**Fig. 3.**
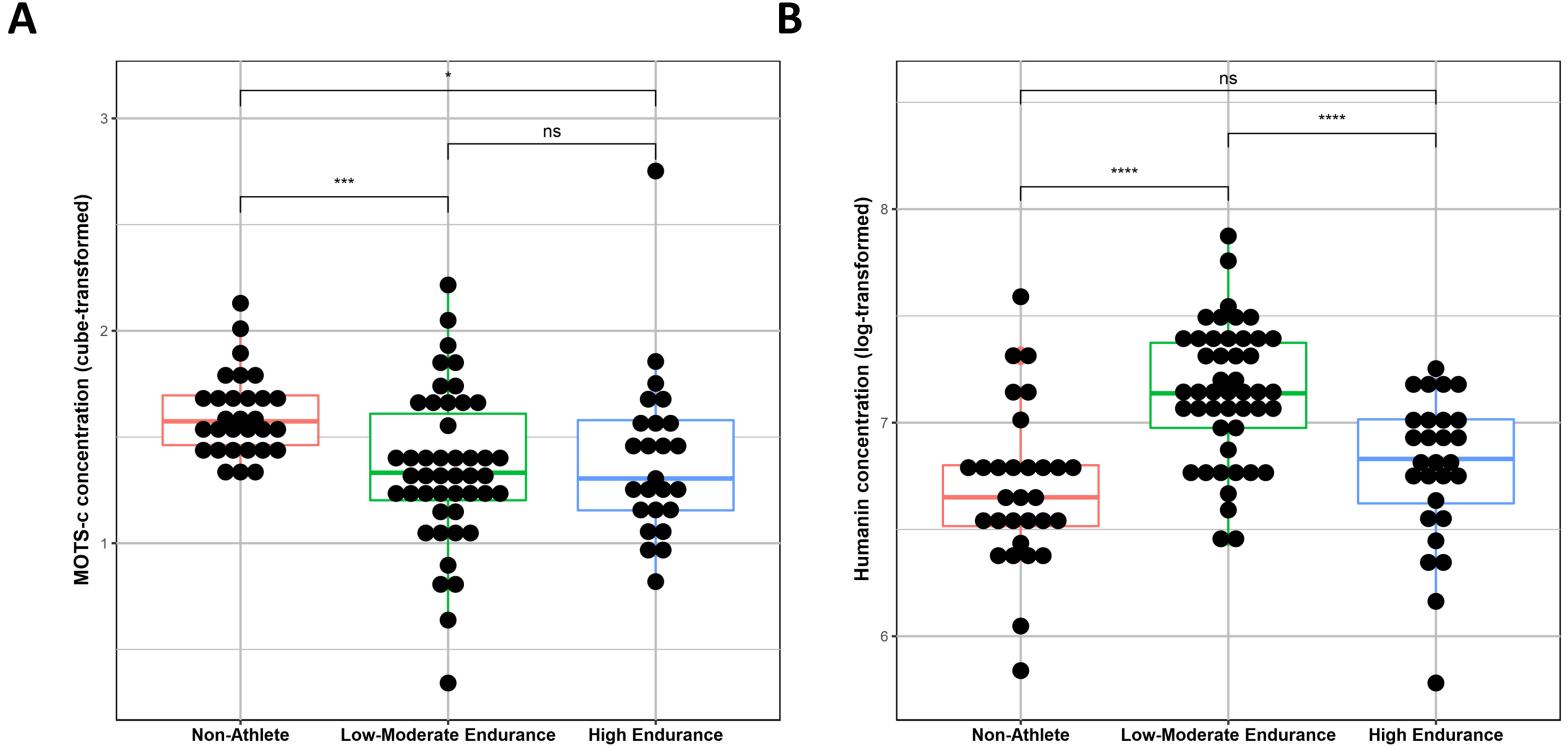
**Differences in MOTS-c (A) and Humanin (B) serum levels among non-athletes, low-moderate endurance athletes, and high endurance athletes**. A linear regression model was generated for both factors serum readings, MOTS-c readings were cube transformed, and humanin readings were log-transformed. Ns = non-significant, *** = *p *< 0.005, **** = *p *< 0.0005.

As we observed a clear difference between the groups, we continued to investigate the levels of Humanin and MOTS-c between non-athlets, low/moderate athletes, and hight endurance athletes. No significant differences in MOTS-c levels were observed between the different athletic groups (Low and High) (Fig. 3A), however, there were significant differences in the MOTS-c levels between the non-athlete and Low/Moderate (*p *= 0.005) and High Endurance groups (*p *= 0.05) (Fig. 3A). On the other hand, Humanin results suggested no significant differences between non-athlete and high endurance groups (Fig. 2B), although a significant difference in Humanin levels was detected between the non-athlete and low-moderate endurance groups (*p *= 0.001), and between the low-moderate and high endurance groups (*p *= 0.0002) (Fig. 3B).

### 3.4 Effect of Age on MOTS-c and Humanin Levels 

**Fig. 4.**
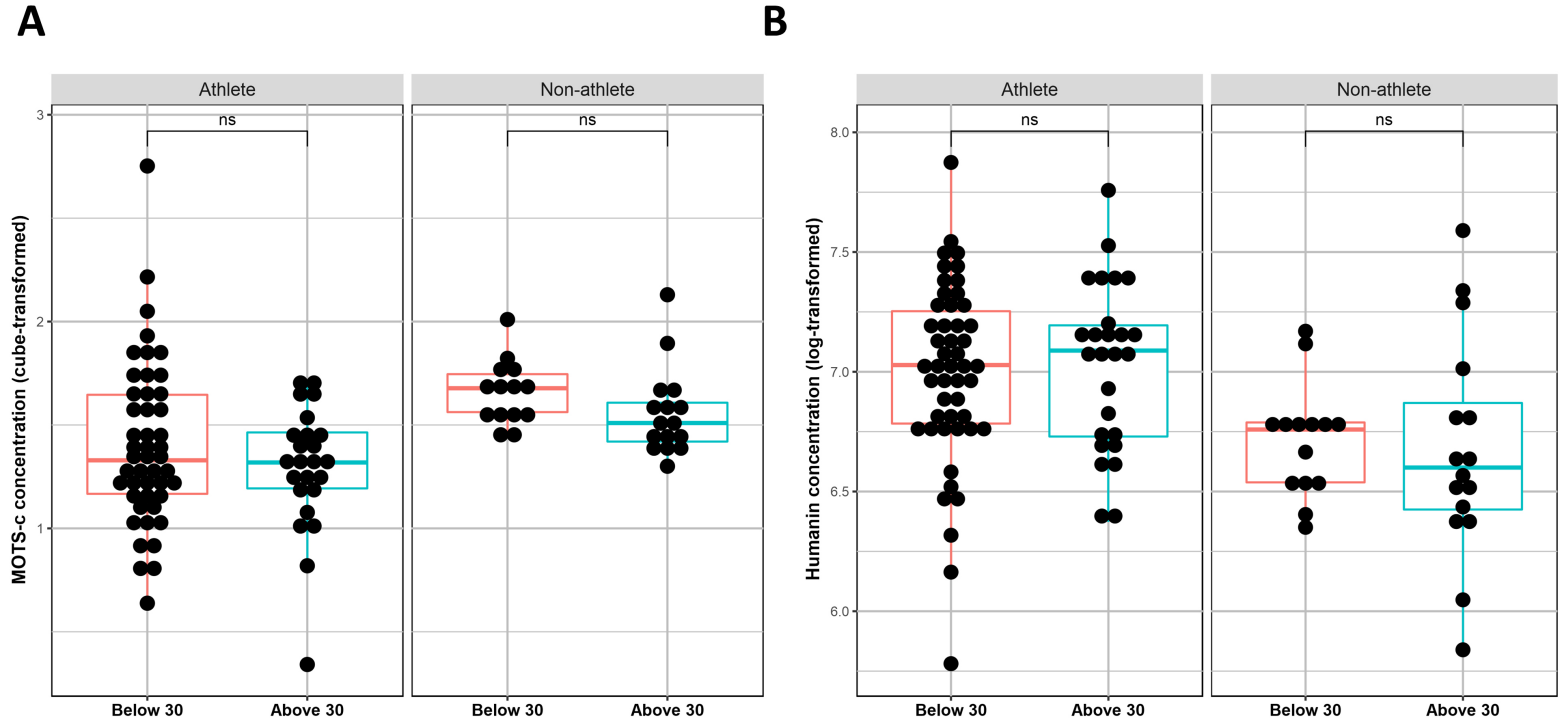
**Differences in MOTS-c (A) and Humanin (B) serum levels between participants above 30 and below 30 in non-athletes and athletes**. The serum levels of 75 athletes (26 above 30 and 39 below 30) and 30 controls (16 above 30 and 14 below 30) were screened for Humanin and MOTS-c. A linear regression model was generated for both factors serum readings. Ns, non-significant.

Since MDPs are known to be affected by age, we compared their expression levels between two age groups in each cohort: above 30 and below 30 years old. Results indicate no significant differences in the expression levels of MOTS-c (Fig. 4A) nor in Humanin (Fig. 4B) between the age groups in athletes or non-athletes.

### 3.5 MOTS-c and Humanin Expression is Positively Correlated

**Fig. 5.**
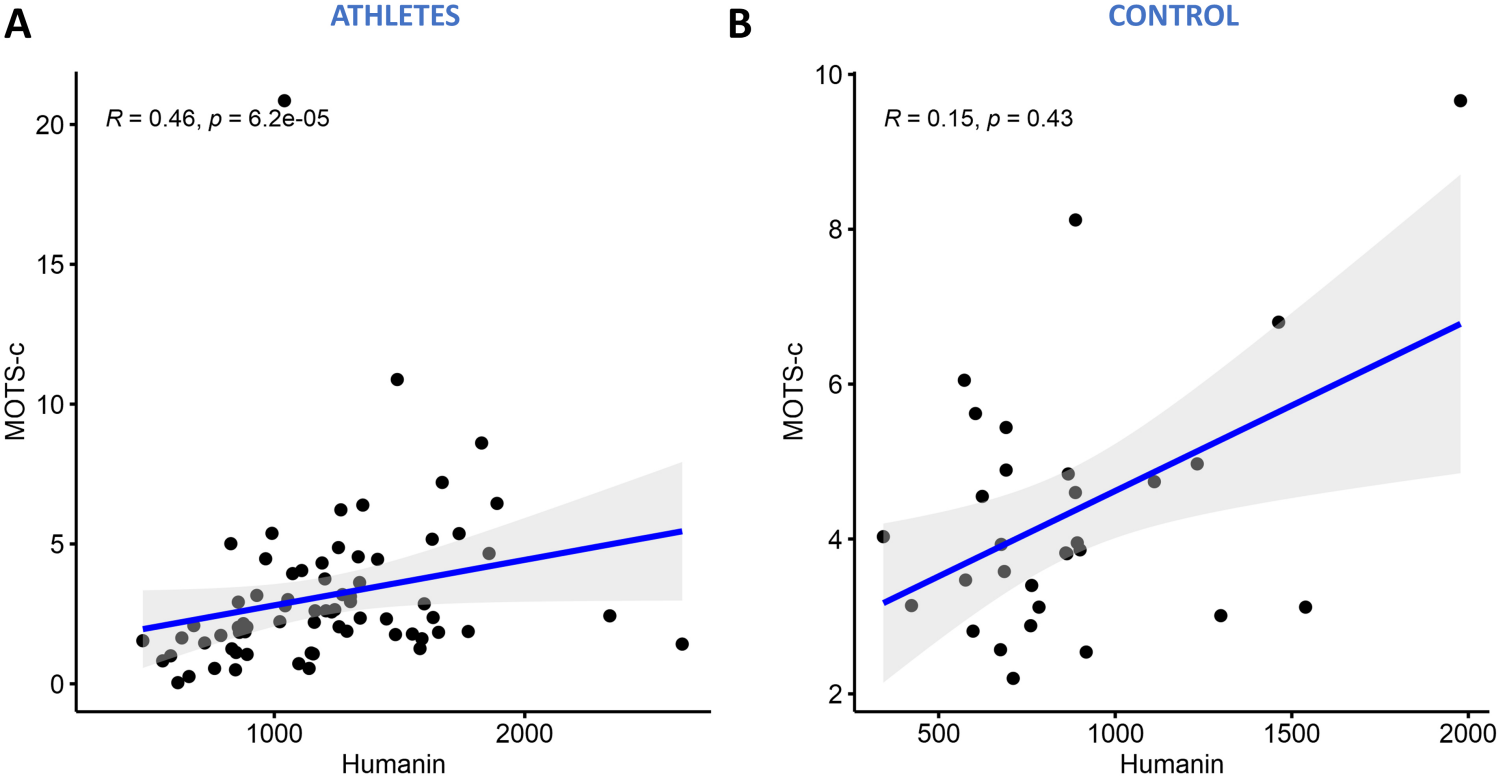
**Spearman’s correlation between MOTS-c and Humanin serum levels**. The correlation test was conducted between athletes (A) and non-athletes (B).

Spearman’s correlation model was used to study the association between Humanin and MOTS-c in athletes and control. Results showed that these Humanin and MOTS-c were significantly correlated in athletic group (R = 0.46, *p* = 6.2 × ${\mathrm{10}}^{-5}$) as shown in Fig. 5A. This correlation was not seen in the control non-athlete group, although the same positive trend was detected (Fig. 5B).

## 4. Discussion

Regular exercise improves health, prevents obesity and its complications such as diabetes. It is also highly associated with improving the health of the cardiovascular system through expression of certain factors/peptides that affect the function of the cardiac muscle and other organs. Therefore, understanding the effect of the exercise on the body at the cellular levels is important. Previous studies have shown that exercising muscle cells exhibit increased mitochondrial count and activity [10]. Studies have also suggested that exercise increases levels of small mitochondrial peptides in both animal models and humans, including the two cytoprotective peptides Humanin and MOTS-c [1, 11, 13]. However, the effect of chronic exercise on the levels of these critical mitochondrial peptides is still not well understood. In this study, levels of MOTS-c and Humanin were assessed in serum samples collected from professional athletes who belong to different sport professions (high and low/moderate endurance sports) and compared between the endurance groups and with sedentary controls (non-athletic cohort).

Recent investigation linked exercise with Humanin levels and health of the cardiovascular system. Principally, oxidative stress is a major factor in multiple CDs as it contributes to apoptosis and fibrosis [16]. Humanin reduces oxidative stress damage in cardiomyocytes as it promotes the expression level of critical oxidative stress pathways’ factors and upregulates the expression of antioxidant enzymes [17]. These effects of Humanin make it a key player in CD generation and progression and a potential therapeutic target for CDs [18]. There is an emerging evidence that shows a link between exercise and Humanin experession level. Mainly, studies have suggested that Humanin levels increase significantly after acute exercise. A recent study has suggested that Humanin levels increase immediately after acute high intensity exercise and 4 hours post-acute exercise in both muscular tissue and plasma in young healthy men. However, the continued high intensity exercise for two weeks did not change Humanin levels in skeletal muscle and plasma [11]. Similarly, the increased plasma level of Humanin after high intensity exercise did not change after acute resistance exercise [19]. Other studies have shown that in metabolically unhealthy cohort, Humanin levels increased after 12 weeks of resistance exercise in skeletal muscle tissue, but not in serum [20]. In our study, we found that Humanin serum levels were highest in low-moderate intensity athletes, reaching 1258 pg/mL in as compared to 774 pg/mL in the controls, while the high intensity cohort had lower levels of Humanin (926 pg/mL). Therefore, our data suggests that the increased Humanin levels in response to chronic exercise, especially in low/moderate endurance athletes, supports data from the acute exercise studies. The long-lasting effect of chronic exercise in professional athletes over Humanin serum concentration may suggest a protective role through reducing inflammation and oxidative stress markers. Humanin data also suggests that low-moderate sustained exercise provides a greater cytoprotective and anti-inflammatory effects than no exercise or high intensity sustained exercise, indicating that severe persistent exercise may not be as beneficial as the lower intensity sustained counterpart. The lack of dose-effect of chronic exercise over Humanin levels may also suggest a dampening of the hypothalamic-pituitary-adrenal axis (HPA axis) reactivity in highly stressed endurance group compared to low/moderate counterpart [21].

Like Humanin, previous studies have suggested that exercise plays a cardioprotective role through MOTS-c. Studies have shown that exercise can improve cardiovascular function and protects against CD via increasing the supply and consumption of oxygen, as well as by reducing the fibrosis and apoptosis in the myocardium [13]. Exercise was also shown to improve survival and function of the cardiomyocytes. Aerobic exercise controls the dynamic of the mitochondria, as well as regulating autophagy, and biogenesis of cardiac hypertrophy [22]. The cardio protective role of MOTS-c has been mostly attributed to improvement of coronary endothelial dysfunction and myocardial remodeling [23], although, the levels of MOTS-c in exercising elite athletes have not been reported. Recent studies reported the link between exercise and MOTS-c. Reynolds *et al*. [1] showed that MOTS-c levels in plasma and skeletal muscle tissue increased after acute high endurance exercise, while MOTS-c plasma levels did not significantly change after resistance exercise. A study was conducted on mice that aimed to study the mechanism of action of MOTS-c. The study found that MOTS-c levels increased after 8 weeks exercise (treadmill running) in both skeletal muscle tissue and serum samples [24]. The increase was seen in both healthy and obese mice, which shows that the different metabolic background does not alter MOTS-c response to acute exercise. Another recent study also showed an increase of MOTS-c post-acute exercise in non-Hispanic white breast cancer patients [25]. Compared to sedentary controls, we observed that MOTS-c levels were significantly lower in professional athletes. Furthermore, mild-moderate athletes exhibited higher levels than athletes who belong to high endurance group. This surprisingly lower concentration of MOTS-c serum levels in professional athletes compared to sedentary controls could reflect an adaptation mechanism associated with chronic endurance exercise, which was further manifested in high endurance group. The functional implications of the reduced MOTS-c levels in professional athletes on cytoprotection, cellular metabolism, glucose homeostasis and insulin sensitivity of skeletal muscle remain to be investigated. However, the strong positive correlation between MOTS-c and Humanin in athletes, but not in sedentary controls, supports the general cytoprotective role of chronic exercise. Interestingly, there were no significant differences in the levels of the two peptides between below and above 30 years old participants in both study groups (athletes and sedentary controls), but our data represent only a snapshot with no longitudinal follow up, hence the effect of age on levels of these peptides cannot be conclusive.

This study is limited by the relatively small size and lack of detailed information about the study participants, including their insulin sensitivity index and lipid profiles. However, our pilot emerging findings will help guide future studies with more controlled longitudinal design to confirm these findings and identify their functional relevance and potential therapeutic applications.

## 5. Conclusions

The cytoprotective mitochondrial peptides MOTS-c and Humanin exhibit different serum concentrations between sedentary and professional endurance athletes who belong to different sport disciplines. Despite their highly correlated levels in athletes, but not in sedentary controls, Humanin shows higher levels in athletes, especially among those who belong to low/moderate endurance group, whereas MOTS-c exhibits lower levels in the athletes groups compared to controls. Future studies investigating the functional implications of these changes are warranted for deeper understanding of the underlying molecular mechanisms associated with different levels of chronic exercise.
